# Exploring the Role of Theory of Mind in Moral Judgment: The Case of Children with Autism Spectrum Disorder

**DOI:** 10.3389/fpsyg.2016.00523

**Published:** 2016-04-18

**Authors:** Roberta Fadda, Marinella Parisi, Luca Ferretti, Gessica Saba, Maria Foscoliano, Azzurra Salvago, Giuseppe Doneddu

**Affiliations:** ^1^Department of Pedagogy, Psychology, Philosophy, University of CagliariCagliari, Italy; ^2^Center for Pervasive Developmental Disorders, Azienda Ospedaliera BrotzuCagliari, Italy; ^3^Studio KaleidosCagliari, Italy

**Keywords:** school children, moral judgment, theory of mind, autism spectrum disorder, moral education

## Abstract

This paper adds to the growing research on moral judgment (MJ) by considering whether theory of mind (ToM) might foster children’s autonomous MJ achievement. A group of 30 children with autism spectrum disorder (ASD) was compared in MJ and ToM with 30 typically developing (TD) children. Participants were tested for MJ with a classical Piaget’s task and for ToM with a second order False Belief task. In the moral task, children were told two versions of a story: in one version the protagonist acted according to a moral intention but the action resulted in a harmful consequence; in the other version the protagonist acted according to an immoral intention, but the action resulted in a harmless consequence. Children were asked which of the two protagonists was the “naughtier.” In line with previous studies, the results indicated that, while the majority of TD participants succeeded in the second order False Belief task, only few individuals with ASD showed intact perspective taking abilities. The analysis of the MJ in relation to ToM showed that children with ASD lacking ToM abilities judged guilty the protagonists of the two versions of the story in the moral task because both of them violated a moral rule or because they considered the consequences of the actions, ignoring any psychological information. These results indicate a heteronomous morality in individuals with ASD, based on the respect of learned moral rules and outcomes rather than others’ subjective states.

## Introduction

The psychological roots of morality have been one of the major focuses of attention of philosophy for centuries. More recently, experimental psychologists empirically investigated moral reasoning in children and adults in the attempt to appreciate the impact of this process on individuals’ social lives.

Piaget (1932) studied the psychological origins of moral judgment in children by using moral dilemmas, in which answers varied along one cognitive dimension (intention) and one contextual dimension (consequence). Piaget (1932) demonstrated that children up to the age of six use consequences rather than intentions to judge culpability in such dilemmas, so they consider the obedience to the moral rule more important than intentions. Piaget called this phase the “heteronomous stage,” in which children consider moral rules as unchangeable and requiring strict obedience. Later in their development, at the end of the school years, children view rules as flexible and related to social principles that vary according to people’s intentions. Piaget named this stage the “autonomous stage,” in which moral transgressions are judged considering the intentions behind the actions (Piaget, 1932).

Subsequent research on children confirmed the role of perspective taking in moral reasoning. The majority of children tend to consider intentional behaviors “wrong” compared to the accidental ones (Nunez and Harris, 1998). The relationship can also run from moral judgment to theory of mind (ToM), so that preschool children judge the intentionality underlying an action depending on the moral valence of the action itself (Leslie et al., 2006a; Pellizzoni et al., 2010). Adults usually rely on mentalistic motivations to define moral judgements about agents’ behaviors. While unintentional behaviors are usually explained merely in terms of mechanical causal factors, intentional actions are interpreted by considering agents’ knowledge that a behavior might cause negative effects on others (Knobe, 2005).

A number of research on TD adults and children indicated the role of perspective taking in moral reasoning. However, as long as perspective taking precedes the development of moral judgment (MJ) in childhood, it is difficult to identify the actual weight of ToM on morality.

Recently, studies of morality in individuals with autism spectrum disorder (ASD) provided important insights into the role of ToM abilities in MJ. Given the mentalizing impairments that characterize individuals with ASD (Baron-Cohen et al., 1985; Baron-Cohen, 1989; Hill and Frith, 2003), it might be predicted that MJ would be impaired in these individuals. Surprisingly, the majority of previous studies showed that individuals with ASD are sensitive to moral transgressions, despite a lack of ToM abilities.

Blair (1996) investigated the ability of children with ASD to distinguish between moral and conventional transgressions. While moral transgressions determine a consequence for the rights and welfare of others, conventional transgressions determine a consequence for the social order. Thus, it is the responsivity to the distress of a victim that is crucial to distinguish moral from conventional transgressions (Smetana et al., 2008). Blair (1996) investigated the responses to the moral/conventional distinction of two groups of children with ASD: one group lacking the ability to mentalize and the other group showing the capacity to mentalize. Typically developing children and moderate learning difficulty children were evaluated as control groups. The results indicate that all the groups made the moral/conventional distinction. Thus, ToM abilities were not crucial for basic MJ.

Leslie et al. (2006b) investigated whether children with ASD make basic MJ in comparison to TD controls in two experiments. In the first experiment, children were tested for very basic MJ about prosocial (“good”) and antisocial (“bad”) acts (Killen, 1991). Participants with ASD were also tested on two standard false belief tasks (Baron-Cohen et al., 1985; Perner et al., 1989). The first experiment indicated that the ASD group, despite a lower ToM performance compared to controls, made simple bi-valued MJs as TD children. The second experiment replicated and extended Blair’s study by introducing a new control task: the “cry baby” story. In this story, the action of the protagonist determined neither a moral nor a conventional transgression. However, the action caused a distress of a baby. Thus, if the participants would simply react to the distress of another person, then they will judge the “cry baby” story as a moral transgression. The same participants with ASD tested in the first experiment were considered (with and without ToM). The results of the second experiment supported the findings of Blair (1996): children with ASD who failed standard false belief tasks distinguished between moral and conventional transgressions. Moreover, both TD and ASD children distinguished between the distress of a “cry baby” and the distress of a victim in a moral transgression. Taken together the results of the two experiments suggest that children with ASD who fail standard false belief tasks may present a basic moral sense.

Lecciso et al. (2008) analyzed the value of the intention behind moral actions and its link with mentalising abilities in high-functioning children with ASD and in normally developing children. The results indicated that, although children with ASD showed difficulties in both first-order and second-order false belief tasks, their ability to make moral judgements was not impaired.

Recently, Kretschmer et al. (2014) investigate relations between moral reasoning, executive functioning and ToM in children with ASD compared to TD children. A dilemma was presented to participants and they had to judge if the protagonist’s behavior was correct or not. In addition, participants completed two ToM tasks, a working memory and an inhibition test. The results indicated that children with ASD did not differ from TD children in cognitive and affective aspects of moral reasoning, ToM and executive functioning. However, there was a correlation between moral reasoning and inhibitory control and between ToM abilities and inhibition. Thus, these results showed that ToM is not a prerequisite for MJ but that inhibitory control might play a mediating role between ToM and MJ in children with ASD.

These studies on MJ in ASD has focused on the ability to distinguish between intentional moral and conventional harms (Blair, 1996). They also focused on the MJ of intentional negative acts as good or bad (Leslie et al., 2006b; Lecciso et al., 2008; Kretschmer et al., 2014). Both kinds of studies found that ASD and TD individuals possess the same basic understanding of moral right and wrong: they can distinguish morally acceptable from morally unacceptable acts.

Other studies applied more complex moral reasoning paradigms, which varied the intentionality of immoral acts requiring participants to involve ToM abilities to make MJ.

Grant et al. (2005) investigated whether children with ASD are able to weight up both the motive and outcome of behavior for judgments of culpability, compared with a group of children with moderate learning difficulties and a group of TD children in MJ. Participants were presented with pairs of stories in which motive and outcomes were combined into three conditions: (A) different motives (ill/good), same outcome; (B) same motives (good), different outcomes (damage to a person/damage to a property); (C) different motives (ill/good) and different outcomes (damage to a person/damage to a property). Participants were asked to judge which protagonist was the naughtier and to justify verbally this judgement. Justifications were analyzed to assess whether correct culpability judgements derived from appropriate, adult-like reasoning, or whether correct judgements did not involve an appreciation of intentions. Results showed that children with ASD were as likely as controls to judge ill intentions more culpability than good ones regardless the outcomes. They judge injury to persons as more culpable than damage to property. Thus, participants with ASD were able to evaluate intentions and outcomes in making MJ. However, no measures of ToM abilities were considered in this study. Moreover, differently from controls, the majority of the justifications given by the children with ASD were reiterations of the story rather than explanations of their judgements.

Moran et al. (2011) tested whether ASD adults would consider both the intentions and the outcomes of a person’s actions when expressing MJ, in comparison to TD controls. Participants read vignettes in which protagonists produced either a negative or a neutral outcome based on the belief that they were causing the negative outcome (negative belief) or the neutral outcome (neutral belief). The MJs required both processing beliefs and intentions and processing outcomes. Participants in both groups were also tested for ToM abilities. The results indicated that there were no differences between participants with ASD and TD controls in ToM abilities. However, adults with ASD showed an over-reliance on the action’s negative outcome and an under-reliance on information about a person’s innocent intention compared to controls. These findings indicate that individuals with ASD don’t integrate mental state information into MJs.

Zalla et al. (2011) investigated the ability of adults with ASD to distinguish moral, conventional and disgust transgressions using a set of six transgression scenarios. Each transgression was followed by questions about permissibility, seriousness, authority contingency, and justification. The results showed that individuals with ASD were able to distinguish between moral and conventional norms. However, they failed to distinguish moral and disgust transgressions and were unable to provide appropriate moral justifications. Moreover, they judged conventional transgressions and disgust transgressions to be more serious compared to TD controls. The seriousness rating correlated with their ToM impairment. The authors concluded that individuals with ASD might be responsive to rule violations, even though they fail to use relevant information about the agent’s intentions in moral reasoning.

Shulman et al. (2012) compared moral and social reasoning in individuals with and without ASD. Familiar schoolyard transgressions were shown to participants and a yes/no question examining the acceptability of the portrayed behaviors was asked to them. They also had to judge the appropriateness of the behavior and explain their judgments concerning acceptability. The judgments were categorized into different categories, corresponding to the Kohlberg’s developmental model of morality. For example, concern over the damage resulting from the specific transgression corresponded to the first stage of pre-conventional level and justifications based on an awareness of general principles prohibiting the depicted behaviors corresponded to the second stage of pre-conventional morality. The results indicated both groups made the moral/social distinction. Participants with TD were more flexible than participants with ASD, providing more examples of behaviors that might be considered as transgressions in one context but not in another one. Moreover, participants with ASD considered more often than TD participants the expected damage which would result from the transgressions and they cited more often a specific simple rule prohibiting such behavior (e.g., “that’s bad,” “you can’t do that”). These results seem to indicate that participants with ASD are at a pre-conventional level of moral development, in which intentions are not yet included in their moral reasoning. However, the authors did not consider differences in ToM abilities between ASD and TD participants. Differences in MJ between groups were explained considering that individuals with ASD focus on the details. Thus, it becomes difficult for them to discern the relevant features (e.g., the intentions) as different and as more important than irrelevant ones (Happé and Frith, 2006).

In summary, previous studies showed that individuals with ASD are able to perform at the same level as TD individuals in basic moral reasoning despite a lack of ToM (Blair, 1996; Leslie et al., 2006b; Lecciso et al., 2008; Kretschmer et al., 2014). Basic moral reasoning tests, which presented participants with a yes/no question to examine the acceptability of an action, have in common that they do not require individuals to explain moral decisions, but only to distinguish whether an action is ‘good’ or ‘bad.’ This might explain the similar performance of individuals with and without ASD.

Other studies revealed differences in moral reasoning between individuals with TD and individuals with ASD when more complex moral reasoning paradigms were applied that put higher demands on reasoning (Grant et al., 2005; Moran et al., 2011; Zalla et al., 2011; Shulman et al., 2012). Specifically, while TD individuals integrate intentions into their justification of moral reasoning, individuals with ASD seems to employ a compensatory strategy, in which the rigid application of a learned moral rule might supply for ToM deficits in this population.

The results of the empirical studies described so far are consistent with the recent philosophical conceptualization of morality in individuals with ASD. Kennet (2002) described individuals with ASD as lacking the ability to self-develop moral rules or to apply them into new situations. According to Kennet’s view, since individuals with ASD have ToM deficits, they fail to represent other people’s thoughts in a particular situation, which is crucial to elaborate a moral judgement. However, individuals with ASD develop moral judgements by other means, like for example by reasoning on explicit rules taught by others and/or by inferring the social norms from past experiences (Kennet, 2002). Krahn and Fenton (2009) recently supported such a view of morality in ASD: individuals with ASD are viewed as not morally autonomous because they lack a sense of intersubjectivity – the ability to understand moral behavior from the viewpoint of an agent involved in an action.

Thus, nowadays philosophers converge in the hypothesis that individuals with ASD should not be considered fully mature moral agents (autonomous) because of their significant social impairment. On the contrary, these individuals express heteronomous MJ based on overlearned abstract knowledge about normative rules (Kennet, 2002; Krahn and Fenton, 2009; Damm, 2010). For the best of our knowledge, this hypothesis of a heteronomous morality in individuals with ASD has never been empirically tested using a developmental model of morality. This model might be helpful to conceptualize MJ in ASD as developmentally immature, rather than merely atypical, whit important implication for interventions.

The only study so far, for the best of our knowledge, that tried to explain morality in individuals with ASD according to a developmental model of morality is Shulman et al. (2012), which indicated that children with ASD tend to express judgments at a pre-conventional stage of Kohlberg’s moral development model. However, this study explained these results in terms of attention to details rather than in terms of ToM abilities, which were not evaluated in the participants. This study left open the question of whether ToM abilities might play a crucial role in the development of a mature level of morality in ASD.

The present study tested the hypothesis of an heteronomous morality in individuals with ASD, in the light of the Piagetian model of moral development in children. Even though Piagetian framework of moral development has been challenged in the last decades, Piaget’s dilemmas might offer an ideal context to verify the hypothesis of a heteronomous MJ in children with ASD, because the answers could vary along intention, transgressions of a moral rule and consequences. To achieve this aim, we investigated ToM and MJ in children with ASD and in typically developing children. We predicted that, if individuals with ASD are heteronomous agents, they will consider more the consequences of the actions or the violations of the moral rule when solving Piaget’s dilemmas rather than the others’ intentions.

## Materials and Methods

### Participants

Two groups participated in the study. The first group included 30 TD school children (all males; average chronological age = 10 years and 6 months; *SD* = 1.687). The TD participants were recruited at school and they were interviewed during the school hours. The second group included 30 participants with ASD (all males; average chronological age = 11 years and 8 months; *SD* = 3.808; average *IQ* = 87.57; *SD* = 17.344). The participants with ASD were diagnosed by expert clinicians using the DSM-IV criteria and Autism Diagnostic Observation Scale (ADOS) scores (Lord et al., 1999). The participants in both groups were all males, considering the higher prevalence of ASD in this gender. Only the IQ scores of the clinical groups were known, but since the TD group consisted of a random group of typically developing children, who were average performing students in mainstream education, the mean IQ of the control group can be considered to be in the normal range. Official authorizations to carry out the research were provided by the school director and the teachers of the classes involved. Informed written consent was obtained from the parents of each participant. The research was conducted in the absence of any commercial or financial relationship that could be construed as a potential conflict of interest. The study was approved by the local ethics committee and carried out in accordance with the Society for Research in Child Development’s (SRCD) Ethical Standards for Research with Children, the Italian Psychological Association’s Ethical Standards for Research with Humans, and the World Medical Association’s Helsinki Declaration, as revised in October 2008.

### Materials

The participants were tested for mentalizing abilities with a second-order FB task (Perner and Wimmer, 1985) and for moral judgment with a classic Piagetian dilemma. The two tasks were presented in a written form. The FB task included also colored pictures, representing the most salient parts of the story.

### Procedure

Participants were tested individually in a quiet room of the school during a school day. For the FB and the moral judgment tasks, the participants were instructed to read the stories carefully and to imagine each hypothetical situation. The experimenter was available for explanation and support. The order of task presentation was counterbalanced between subjects.

#### Second-Order False-Belief Attribution Task

In the second-order FB attribution task (Perner and Wimmer, 1985), the children were told a story in which two characters, A and B, saw an ice-cream van in the park. Later, each was independently told that the ice-cream van had moved from the park to the church, but they did not know that the other one had also been informed. The participants were asked where A thought B would go to buy ice cream. To respond correctly, the children must employ recursive thinking about mental states, by predicting one person’s thoughts about another’s thoughts. Specifically, the participants should take into account A’s ignorance of B’s knowledge of the true location of the ice-cream van. The second-order FB question was administered only if the child passed a memory question and a reality question. The belief question was scored in a pass or fail manner (Baron-Cohen, 1989). Specifically, the answer was considered correct if the child considers the false belief in order to predict the behavior of the protagonist. On the other side, the answer was considered incorrect if the child considers the real facts to predicts the behavior of the protagonist.

#### Moral Judgment Task

Moral judgment was assessed by telling children the following stories, in which two protagonists used the scissors without the supervision of an adult (transgression):

(a) Margherita wants to play with the scissors when her mum is gone. As soon as she plays with the scissors she cut a little hole in her dress.
(b) Lucia wants to please her mother with a little present. She decides to cut some flowers from a colored paper sheet and give them to her mum. While she’s doing her job, she cut a big hole in her dress.

In the first version of the story, the character intentionally acted unfairly but she caused an insubstantial material damage. In the other version, the character acted fairly but she caused a considerable material damage. The participants were asked to decide which of the two protagonists was naughtier and why. An experimenter, which was blind to the group from which the responses were drawn, judged the participants’ answers. The responses were coded as follows: intention – the child refers to the intention of the protagonist (e.g., “Margherita was the naughtier because she used the scissors just for fun); transgression of a moral rule (moral rules)- the child considers both protagonist as equally nasty (e.g., “Both children were naughty because they used the scissors without the supervision of an adult); consequence – the child refers to the consequence of the action (e.g., “Lucia was the naughtier because she made a bigger hole in her dress compared to Margherita”). While the answers using the intentions indicate the autonomous stage, the other two categories indicate an heteronomous stage.

#### Design and Analysis

A series of non-parametric analysis were applied to compared ASD children and TD children in MJ and ToM. Chi-square was used to compare group differences in ToM and MJ in the general sample. Moreover, chi-square was used to compare group differences in MJ in two subgroups of participants, with and without ToM abilities. Finally, Fisher’s exact test was applied to compared the frequency of participants that expressed heteronomous vs. autonomous MJ in the participants with and without ToM abilities.

## Results

The results indicated that, while half of the TD participants succeeded in the second-order FB task (50%), only a few children with ASD (5%) showed intact perspective-taking abilities (χ^2^ = 7.5; df = 1; *p* = 0.006).

**FIGURE 1:**
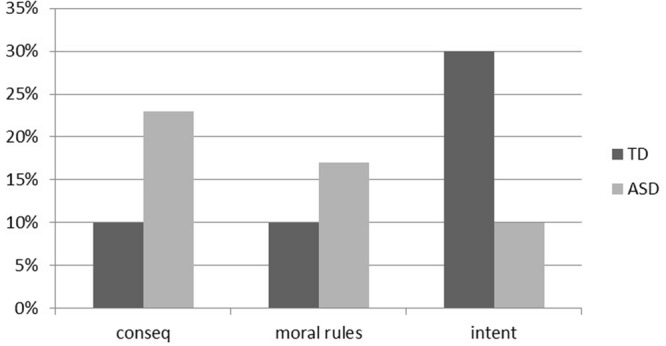
**Percentage of participants with autism spectrum disorder (ASD) and with typical development (TD) who expressed a judgment of consequence (conseq), transgression of a moral rule (moral rules) and intention (intent) in the Piaget’s moral judgment (MJ) task**.

As shown in **Figure 1**, children with ASD judged the culpability on an action mainly in terms of consequences (23%) and transgression of a moral rule (17%). Only a few of the children with ASD referred to the intentions (10%). On the opposite, TD children mainly referred to the intentions (30%) and only a few of them considered the consequences (10%) and the transgression of a moral rule in their judgment (10%). The differences between group were statistically significant (χ^2^ = 10.200; df = 2; *p*= 0.006).

**FIGURE 2:**
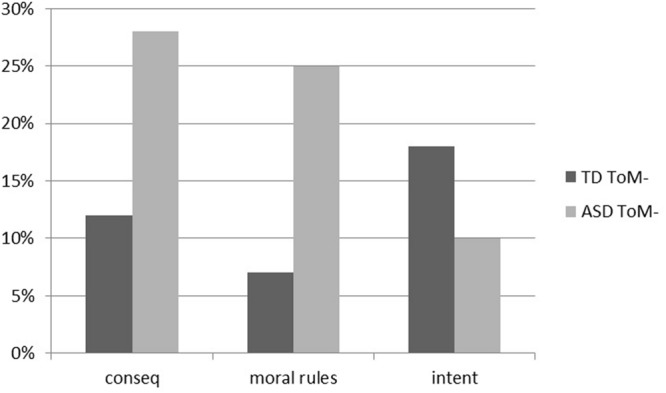
**Percentages of participants with typical development that failed the theory of mind task (TD ToM-) and percentages of participants with autism spectrum disorder that failed the theory of mind task (ASD ToM-) which expressed a MJ according to the intention (intent), the transgression of the moral rule (moral rules) and the consequences (conseq) of the protagonists**.

Then, we considered the MJ in children with TD and ASD divided into two subgroups of participants, with and without ToM abilities. In the subgroup of participants that failed the False-Belief Task (**Figure 2**), the results indicated that, even though the differences between groups are not statistically significant (χ^2^ = 4.627; df = 2; *p* = 0.099), there is a tendency in TD children to consider the intentions of the participants (18%). On the opposite, children with ASD tend to consider mainly the consequences (28%) or the transgression of the moral rules (25%).

In order to identify the heteronomous participants, we collapsed in a unique group the participants that considered the consequences and the transgression of the moral rules in the MJ and we compared them to the autonomous participants, who considered the intentions (**Table 1**). The results indicated that there is a tendency in children with ASD lacking ToM to be mainly heteronomous agents, while TD children tend to be mainly autonomous agents (Fisher’s exact test *p* = 0.06).

**Table 1:** Frequencies of participants with autism spectrum disorder that failed the theory of mind task (ASD ToM-) and typical development that failed the theory of mind task (TD ToM-) that showed heteronomous or autonomous MJ.

	Heteronomous	Autonomous
TD ToM - (*n* = 15)	8	7
ASD ToM - (*n* = 25)	21	4

**FIGURE 3:**
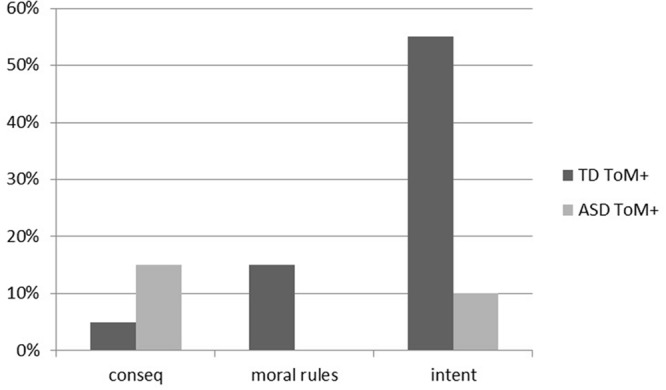
**Percentages of participants with typical development that succeeded in the theory of mind task (TD ToM+) and percentages of participants with autism spectrum disorder that succeeded in the theory of mind task (ASD ToM+) which expressed a MJ according to the intention (intent), the transgression of the moral rule (moral rules) and the consequences (conseq) of the protagonists**.

When we considered the participants that passed the ToM task (**Figure 3**), TD children mainly considered the intentions (55%). Only few children with ASD passed this task and they were distributed between those that considered the intentions (10%) and those that considered the consequences (15%). The differences between groups are statistically significant (χ^2^ = 6.97; df = 2; *p* = 0.031).

As shown in **Table 2**, the results indicated that, even though the differences between groups are not statistically significant (Fisher’s exact test *p* = 0.2898), there is a tendency in TD children to be mainly autonomous agents.

**Table 2:** Frequencies of participants with autism spectrum disorder that succeeded in the theory of mind task (ASD ToM+) and with typical development that succeeded in the theory of mind task (TD ToM+) that showed heteronomous or autonomous MJ.

	Heteronomous	Autonomous
TD ToM + (*n* = 15)	4	11
ASD ToM + (*n* = 5)	3	2

## Discussion and Conclusion

In line with previous studies, our results indicated that children with ASD develop a MJ, despite their deficit in ToM. However, children with ASD who lacked ToM abilities showed more negative MJ concerning actions that broke the rules of morally appropriate behavior or regarding damaging outcomes. Reduced ToM abilities in children with ASD seem to enhance their likelihood to conform rigidly to a social norm or to consider the outcomes of an action, which is typical of a heteronomous stage of moral development in the Piagetian model. Thus, the results of our study confirm our hypothesis of a heteronomous morality in individuals with ASD. Our results are also consistent with a recent philosophical conceptualization of morality in individuals with ASD. As described in the introduction, philosophers nowadays agree that individuals with ASD should not be considered fully mature moral agents due to their social deficits (Kennet, 2002; Krahn and Fenton, 2009; Damm, 2010). These results indicate, as Kohlberg (1971) already said in his studies, that philosophy and psychology can significantly enrich each other in achieving new knowledge in the field of morality.

A possible explanation for these results might be that individuals with ASD reach the heteronomous stage of moral development thanks to their experiences, which promotes the learning of moral rules developed by other people, but fail to advance to the autonomous stage due to their ToM deficits. Similar to the morally heteronomous TD children, children with ASD seem to have an immature sense of morality, so they view moral rules as unchangeable and requiring strict obedience. According to the Piaget’s model of moral development, the developmental transition from a heteronomous morality to an autonomous one might be promoted by the metarepresentational ability to consider others’ viewpoints (Piaget, 1932). Thus, the results of the present study emphasize the importance of evaluating ToM in children with ASD, indicating that interventions aimed at improving children’s MJ need to take theory-of-mind abilities into consideration as a possible pivotal ability. It might be of interest to investigate the possible effect of ToM training on moral reasoning in children with ASD in a future intervention study.

A possible alternative explanation for our results might be that executive functions (EFs) also play a key role in moral judgement, considering the atypical inflexibility in morality expressed by individuals with ASD. Indeed, EFs have been shown to be impaired in young individuals with ASD and ToM deficits but not in the TD control group in whom the two abilities were found to be independent (Ozonoff et al., 1991). Moreover, a recent study found a correlation between moral reasoning and inhibitory control in school children with ASD (Kretschmer et al., 2014). Thus, in future studies, it might be of interest to investigate whether possible prefrontal impairments in ASD, which are capable of causing dysfunctions in a wide variety of neuropsychological domains, such as ToM and EF, might also account for rigid moral judgements in ASD.

This study has some limitations that need to be acknowledged. First, participants were asked to read the second order false belief vignette, which is a rather complex story, so that participants may fail this task just by a lack of motivation or basic understanding. However, as we explained in the procedure, the second-order FB question was administered only if the child passed a memory question and a reality question. These questions allowed to control for possible intervening effects of a lack of motivation or basic understanding in the participants performance (Perner and Wimmer, 1985). Moreover, we decided to test ToM abilities with the “ice-cream van story” rather than a different version of the test, the so-called ‘birthday puppy story’ (Sullivan et al., 1994), for several reasons. The “birthday puppy story” has been shown to elicit better performance in TD children, thanks to a simplified procedure (Hayashi, 2007) and an additional question about the content of the belief (Miller, 2012). However, its wording is more complex than “the ice-cream van” story, and it includes deception, whose beneficial effects on second-order false belief understanding are still controversial (Miller, 2012). Given that individuals with ASD are known to have difficulties with deception (Russell et al., 1991) and with the understanding of language (Volkmar et al., 2005), we decided to administer the Perner and Wimmer’s task to reduce the possible effect of language and deception. Moreover, considering that individuals with ASD respond most favorably to information that is presented visually (see for example Ganz, 2007), we presented the FB task with colored vignettes representing the most salient parts of the story, to aid participants’ understanding and motivation.

A second limitation of our study might be that we used only one task to assess perspective taking. Since the use of only one task has been identified as a methodological limit in previous studies on ToM (see for example, Valle et al., 2015), it might be of interest to use in a future study more different tasks to assess perspective taking, to better define participants mentalistic abilities. Moreover, we used laboratory tasks, which seem to enhance the abilities of children with ASD to reason about social contents (see for example, Sally and Hill, 2006). Thus, it would be interesting in the future to further investigate ToM and MJ in more ecological settings, such as daily life situations.

## Conclusion

The results from the present study lead to the need of important intervention strategies which might help individuals with ASD understand and reason about social interactions in an effective way. Rather than simply teach the “immorality” of an action, new programs should teach the mentalistic principles upon which moral rules are based, and stress the psychological motives of the behavior. It might be of interest, in a future study, to investigate the effect of a training in ToM abilities on autonomous MJ in individuals with Autism Spectrum Disorder.

## Author Contributions

RF contributed to the conception and design of this work. She collected, analyzed and interpreted the data. She wrote the manuscript. MP made the conception and design of this work. She contributed to the analysis and interpretation of the results. LF, GS, MF, and AS contributed to data collection, to the analysis and interpretation of the results. GD contributed to the conception and design of this work and to the interpretation of the data. All the authors reviewed the final version of the manuscript and approved it for publication.

## Conflict of Interest Statement

The authors declare that the research was conducted in the absence of any commercial or financial relationships that could be construed as a potential conflict of interest.
